# Flexible Metal/Polymer Composite Films Embedded with Silver Nanowires as a Stretchable and Conductive Strain Sensor for Human Motion Monitoring

**DOI:** 10.3390/mi10060372

**Published:** 2019-06-04

**Authors:** Jinjin Luan, Qing Wang, Xu Zheng, Yao Li, Ning Wang

**Affiliations:** Institute of NanoEngineering, College of Civil Engineering and Architecture, Shandong University of Science and Technology, Qingdao 266590, China; 17860775202@163.com (J.L.); zhengxu081@163.com (X.Z.); liyaozcx@163.com (Y.L.); wn19861125@163.com (N.W.)

**Keywords:** wearable strain sensor, mechanical property, conductivity property, silver nanowires, human motion monitoring

## Abstract

To avoid conductive failure due to the cracks of the metal thin film under external loads for the wearable strain sensor, a stretchable metal/polymer composite film embedded with silver nanowires (AgNWs) was examined as a potential candidate. The combination of Ag film and AgNWs enabled the fabrication of a conductive film that was applied as a high sensitivity strain sensor, with gauge factors of 7.1 under the applied strain of 0–10% and 21.1 under the applied strain of 10–30%. Furthermore, the strain sensor was demonstrated to be highly reversible and remained stable after 1000 bending cycles. These results indicated that the AgNWs could act as elastic conductive bridges across cracks in the metal film to maintain high conductivity under tensile and bending loads. As such, the strain sensor engineered herein was successfully applied in the real-time detection and monitoring of large motions of joints and subtle motions of the mouth.

## 1. Introduction

Electronic devices that can register physical signals from human body movements in real time are considered to be essential for various applications such as artificial muscles [1], wearable electronics [2,3] and human–machine interfaces [4,5]. For these applications, high sensitivity, good durability, and a large operating range of the device (or sensor) are indispensable features, which allow the sensor to be attached directly to the skin or clothing for monitoring various human movements [6]. In addition, achieving both excellent mechanical property and stable conductivity property is essential toward the application of wearable strain sensors.

Most recently, several conductive materials, including metal thin films [7,8,9], carbon nanotubes [10,11], metal nanowires [12,13,14], and graphene [3,15,16], have been deposited on flexible substrates to fabricate strain sensors. However, despite these recent advances, there are still challenges to overcome in the fabrication of effective tensile strain sensors that are suitable for monitoring complex human motions. For instance, metal/polymer-based strain sensors typically exhibit microcracks because of strain localization and necking effects of the metal thin film under external loads [9]. These cracks limit the application of metal thin film strain sensors to typically monitoring microstrains [17,18]. To achieve stable conductivity under large strains, metal films have been typically deposited on “wrinkled” substrates, which are obtained by pre-stretching [7,19,20], and solvent induction [21] and heat induction [22,23] technologies. Though these methods could be successfully applied to produce wrinkles on the substrate and reduce the number of cracks on the metal layer when the strain sensor is subjected to tensile and bending loads, the sensitivity was low before the wrinkles failed (unfolding with the external loads). Therefore, this illustrates that wrinkled composite materials are unsuitable as wearable strain sensors. Moreover, the conductivity of the strain sensor declined considerably owing to cracks that formed when the wrinkles invalided under the large strain. Considering the excellent conductivity and flexibility due to the high length-diameter ratio of AgNWs [6,24,25], the objective of this work is to avoid conductive failure due to the cracks of metal thin film under tensile and bending loads for the wearable strain sensor by embedding the AgNWs into the Ag film. Furthermore, we aim to obtain high sensitivity and durability for its application in monitoring the motions of the elbow, finger, and mouth.

The strain sensor was fabricated based on a flexible metal/polymer composite film embedded with AgNWs by dip-coating and depositing techniques. The microstructural and changes in the conductivity of the sensor during stretching were thoroughly investigated. The reversibility and stability of the sensor were examined under a tensile strain of 30% and repeated bending loads. Owing to its excellent mechanical property and stable conductivity property, the application of the sensor as a wearable strain sensor was assessed directly by attaching the sensor to various parts of the human body.

## 2. Experimental Section

### 2.1. Materials and Characterization

Polydimethylesiloxane (PDMS) precursor and PDMS curing agent were purchased from Dow Corning SYLGARD (Midland, MI, USA). A AgNWs (5 mg/mL) solution dispersed in ethanol was obtained from Jining LeaderNano (Jining, China). The average diameter and length of the AgNWs were 40 nm and 10 μm, respectively. The AgNWs dispersion was used directly without removing the polyvinyl pyrrolidone that caps the nanowires. The purity of the silver target was 99.9%.

Scanning electron microscopy (SEM; Hitachi S-4800, Tokyo, Japan) images were obtained to study the morphology features of the films. The resistance of the strain sensor was measured using a multimeter (UT890C+, UNI-T, Dongguan, China).

### 2.2. Device Fabrication

**Figure 1:**
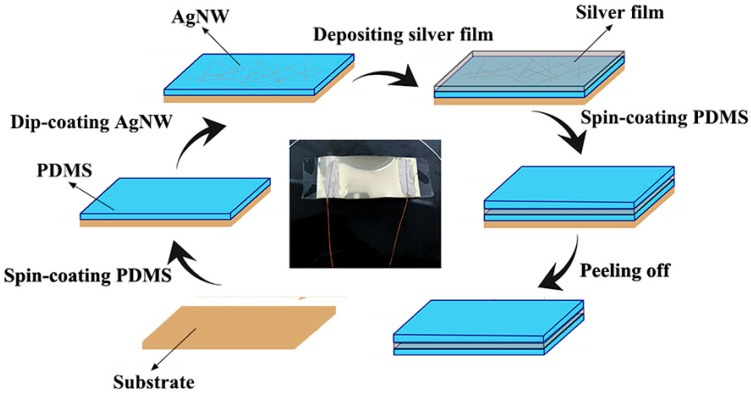
Schematic illustration of the fabrication of the strain sensor.

Figure 1 illustrates the preparation process of the strain sensor based on a metal/polymer composite film by dip-coating and depositing techniques. First, a polycarbonate (PC) substrate was cleaned by absolute ethanol and deionized water. Subsequently, a mixed solution of PDMS prepolymer and curing agent with a weight ratio of 10:1 was spin-coated on the PC substrate at 500 rpm for 40 s, followed by curing at 80 °C for 3 h in a vacuum drying oven. The cured PDMS film was then dip-coated in a diluted AgNWs ethanolic dispersion (0.5 mg/mL) for three times to obtain a uniform coating on the PDMS film, followed by drying at 80 °C for 20 min until the ethanol evaporated. A 100 nm thick Ag film was then deposited on the AgNWs layer through vacuum thermal evaporation at an evaporation rate of 8 nm/s. The Ag/PDMS sensor was fabricated by depositing a 100 nm thick Ag film on the cured PDMS film under the same experimental conditions. The silver paste was used to bond two copper wires to both ends of the device for electrical characterization. A thin PDMS film was spin-coated on the surface of the Ag film to prevent oxidation of the metal film and improve the stability and durability of the device [26]. Finally, the Ag/AgNWs/PDMS and the Ag/PDMS strain sensors were obtained by peeling off the composite film from the PC substrate; a photograph of the Ag/AgNWs/PDMS strain sensor is shown in Figure 1.

## 3. Results and Discussion

### 3.1. Morphology of the Ag/AgNWs/PDMS Composite Film

**Figure 2:**
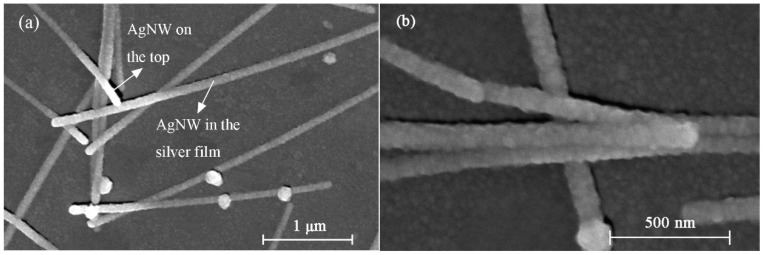
(**a**) Low- and (**b**) high-magnification scanning electron microscopy (SEM) images of the strain sensor showing the Ag film and silver nanowires (AgNWs).

Representative top-view SEM images of the Ag/AgNWs film on the PDMS substrate are shown in Figure 2. As observed in Figure 2a, the “smooth” areas between the AgNWs corresponded to the deposited Ag film. In addition, most of the AgNWs were seen to be embedded within the Ag film, while only the junctions of intersecting nanowires were exposed on top of the Ag film. Figure 2b shows the junctions in the AgNWs network. The formation of these junctions was attributed to evaporation of the solvent during the heating process, which instigated the bonding of neighboring AgNWs. Furthermore, Ag particles were observed around the exposed AgNWs, which are expected to improve the conductivity and decrease NW–NW contact resistance by increasing the contact surface area. In metal thin film strain sensors, external loads, such as tension and bending, are the main factors that compromise the conductivity of the sensor, owing to the formation of cracks in the metal film. The AgNWs are expected to operate as elastic conductive bridges which could help maintain the conductivity of the strain sensor under tensile and bending loads.

### 3.2. Tensile Property

**Figure 3:**
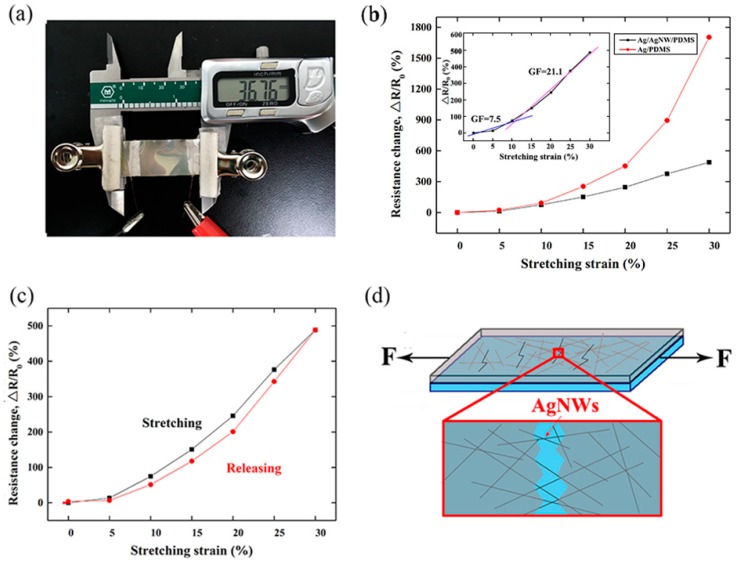
(**a**) Photograph of the experimental setup for measuring the relationship between Δ*R*/*R*_0_ and applied tensile strain. (**b**) Δ*R*/*R*_0_ profile of the Ag/PDMS sensor and Ag/NWs/PDMS strain sensor as a function of tensile strain. Inset shows the Δ*R*/*R*_0_ profile and associated GFs of the Ag/AgNWs/PDMS strain sensor in response to an applied tensile strain. (**c**) Δ*R*/*R*_0_ profile of the Ag/AgNWs/PDMS strain sensor under 1 stretch–release load cycle. (**d**) Schematic diagram of the conductivity principle of the Ag/AgNWs/PDMS strain sensor.

Figure 3a shows the setup used to measure the relative change of resistance (Δ*R*/*R*_0_) in the sensor under different tensile strain. The sensor was secured to the vernier caliper by two insulating jigs and the appropriate strain was achieved by moving the vernier caliper. The resistance was measured by the multimeter, which was connected to the alligator clip and the copper wires. Figure 3b shows the Δ*R*/*R*_0_ profile of the strain sensor under different tensile strains (0, 5, 10, 15, 20, 25, and 30%). The measured *R*_0_ values of the Ag/PDMS sensor and the Ag/AgNWs/PDMS strain sensor were 8.6 and 5.9 Ω, respectively. Under a strain of 30%, the Δ*R*/*R*_0_ of the Ag/PDMS sensor increased to 17.03, which was considerably higher than that of the Ag/AgNWs/PDMS strain sensor (4.88). Notably, within the range of strain examined, the Δ*R*/*R*_0_ of the Ag/PDMS sensor increased to a greater extent than that of the Ag/AgNWs/PDMS strain sensor with increasing strain, indicating that the Ag/AgNWs/PDMS strain senor possesses better conductive stability than the Ag/PDMS sensor. The fast-growing Δ*R*/*R*_0_ of the Ag/PDMS sensor was attributed to cracks in the Ag film under the large strain [9]. Furthermore, the Δ*R*/*R*_0_ curve of the Ag/AgNWs/PDMS strain sensor featured two slopes in two different ranges of strain as shown in the inset of Figure 3b. The strain sensor displayed a moderate gauge factor (GF) of 7.5 when the strain (*ε*) reached 10%. When the strain increased to 30%, GF increased to 21.1. The wrinkled gold thin film critically limited strain sensitivity (gauge factor (GF) < 0.2) even at large tensile strain [22]. The change in conductivity (P/P_0_) of the conductive film by prestrained polyelectrolyte nanoplatforms was only 10% when the tensile strain went up to 70% [19]. This restricts its applicability as a wearable sensor in monitoring subtle movements. The Δ*R*/*R*_0_ of the strain sensor under cyclic tensile strain was also evaluated as shown in Figure 3c. When the strain was reduced from 30 to 0%, only a slight hysteresis was observed with the Δ*R*/*R*_0_ increased by 3.39% relative to the initial value owing to the cracks in the Ag film and the irreversible deformation of the AgNWs. These results indicate that the Ag/AgNWs/PDMS strain sensor is highly reversible with minimal hysteresis during the process of stretching–releasing within a strain of 0–30%. Additionally, the results in Figure 3b,c indicate that the strain sensor has more conductive paths (i.e., AgNWs) than the Ag/PDMS sensor, which can serve as elastic conductive bridges within the Ag film under tensile load. This is due to the excellent flexibility and conductivity displayed by the strain sensor, as schematically depicted in Figure 3d.

### 3.3. Bending Property

**Figure 4:**
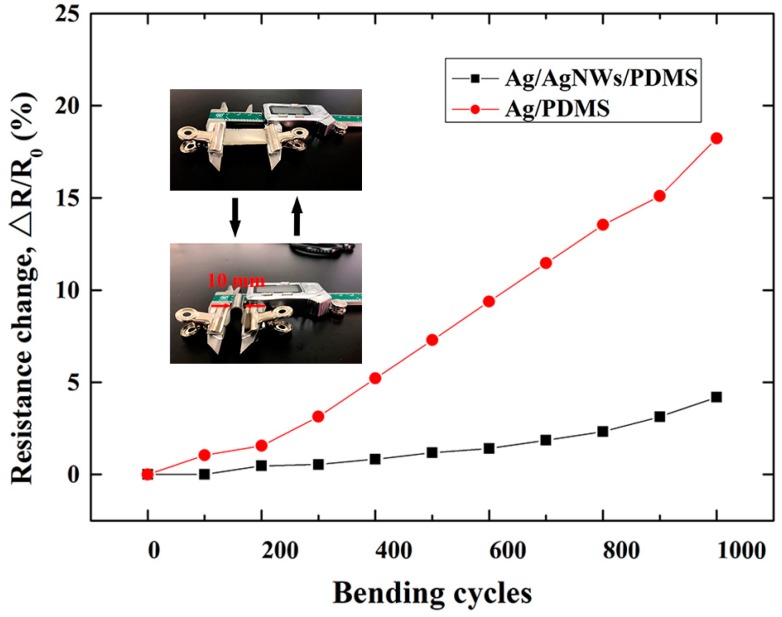
Δ*R*/*R*_0_ profiles of the Ag/PDMS sensor and Ag/AgNWs/PDMS strain sensor under repeated bending cycles with a bending radius of 0.5 cm.

For wearable strain sensors, the bending property of the device also plays a key role in its performance. Figure 4 shows the Δ*R*/*R*_0_ profiles of the Ag/PDMS sensor and Ag/AgNWs/PDMS strain sensor that were subjected to cyclic bending (bending radius of 0.5 cm). Δ*R*/*R*_0_ values were measured at every 100 cycles for up to 1000 cycles. The Δ*R*/*R*_0_ of the Ag/PDMS strain sensor increased to 18.23% after 1000 bending cycles. In contrast, the Δ*R*/*R*_0_ of the Ag/AgNWs/PDMS strain sensor increased to approximately 4% after 1000 bending cycles. The AgNWs merely coated on the polyurethane acrylate (PUA) could not maintain their conductivity for more than 600 cycles [27]. This result indicates that the Ag/AgNWs/PDMS strain sensor has good bending fatigue strength. Furthermore, the bonding of the AgNWs plays an important role in contributing to the high bending performance and durability of the strain sensor. The significant reduction in conductivity of the Ag/PDMS was attributed to cracks of the Ag film and fewer conductive paths when compared with the Ag/AgNWs/PDMS strain sensor.

### 3.4. Human Motion Monitoring

**Figure 5:**
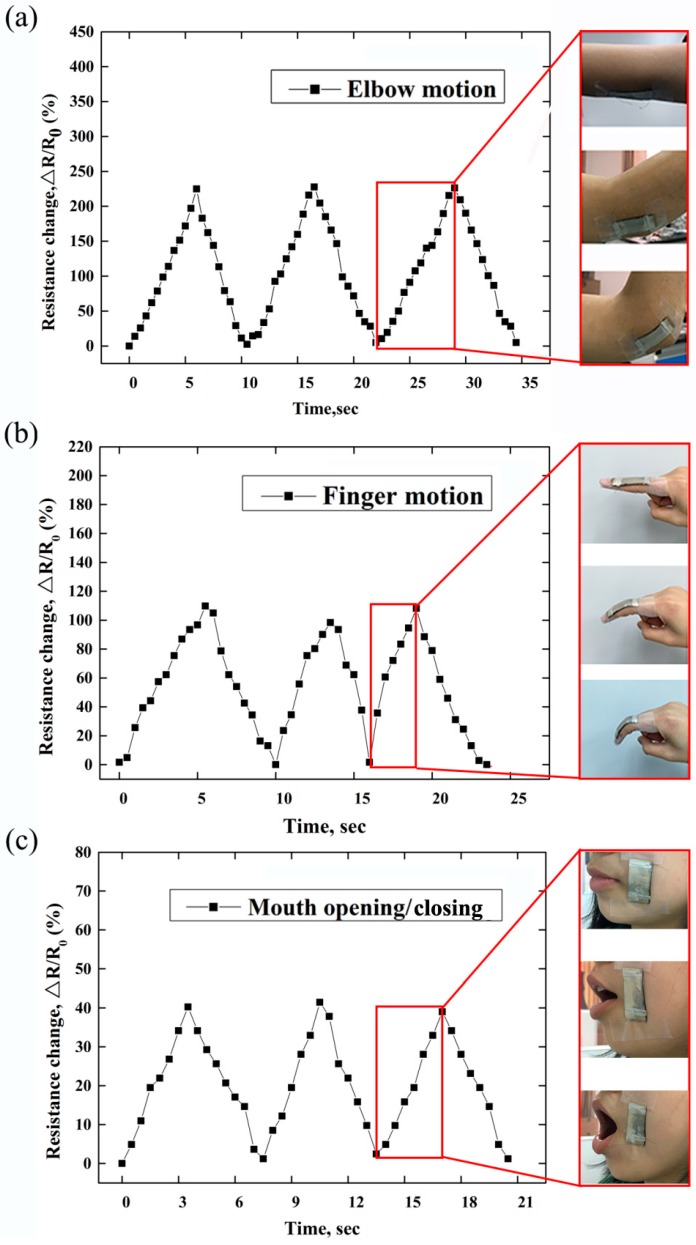
Δ*R*/*R*_0_ profiles of the stain sensor under motions of the (**a**) elbow, (**b**) finger and (**c**) mouth.

To illustrate the applicability of the Ag/AgNWs/PDMS strain sensor for monitoring human body movements, the strain sensor was connected to different parts of the body and the Δ*R*/*R*_0_ was measured under the movement of various muscles. The three illustrations in the red frame pointed by the single peaks of the resistance change are the corresponding movements of the elbow, index finger and mouth. Figure 5a,b shows the Δ*R*/*R*_0_ curves when the strain sensor was connected to the elbow and index finger joint as they performed continuous bending–straightening motions. As observed in Figure 5a, when the elbow was bent, the sensor responded promptly, as indicated by the peaks in the Δ*R*/*R*_0_ profile (as high as 220%), owing to stretching of the strain sensor. Similarly, rapid responses were recorded when the finger carried out repetitive bending–straightening motions (Figure 5b). The Δ*R*/*R*_0_ increased as the finger joint had bent, reaching a maximum value of 110% when the finger was bent to the maximum. In addition, the strain sensor was attached directly to the face to monitor the movements of the facial muscles. Likewise, as observed in Figure 5c, the sensor promptly and repeatedly produced Δ*R*/*R*_0_ signals during the repeated mouth opening–closing processes. More importantly, the Δ*R*/*R*_0_ increased during the process of elbow bending, finger bending, and mouth opening and returned to its initial value after each cycle of motion, owing to the high reversibility of the strain sensor. These findings demonstrate that the Ag/AgNWs/PDMS strain sensor has good stability and reversibility features that are necessary for the application of a wearable strain sensor for monitoring human movements such as joint and facial movements.

## 4. Conclusions

A highly sensitive, reversible and stable strain sensor based on a flexible metal/polymer composite film embedded with AgNWs was successfully fabricated by dip-coating and depositing techniques. The Ag/AgNWs/PDMS strain sensor demonstrated high sensitivity with GFs of 7.5 at lower strain (≤ 10%) and 21.1 at higher strain (10–30%). In addition, the strain sensor exhibited high reversibility (Δ*R*/*R*_0_ ± 3.39%) in a relatively wide working (strain) range of 0–30%. Moreover, the Δ*R*/*R*_0_ of the Ag/AgNWs/PDMS strain sensor increased by only 4.19% after 1000 bending cycles relative to the initial value, thus, highlighting the excellent stability and durability of the sensor. These results indicated that the AgNWs could act as elastic conductive bridges across cracks in the metal film to effectively avoid conductive failure under external loads of the Ag/AgNWs/PDMS strain sensor. Based on such desirable features, the sensor was successfully applied in the real-time detection of a wide range of human activities from large joint motions to subtle facial movements. The excellent mechanical property and stable conductivity of the Ag/AgNWs/PDMS strain sensor illustrate its potential as a wearable device with a large monitoring range.
